# Deciphering the Ubiquitin-like Code of DNA-PK: Mechanisms and Therapeutic Opportunities

**DOI:** 10.3390/biom16040498

**Published:** 2026-03-26

**Authors:** Jiaqi Zhao, Zhendong Qin, Jiabao Hou, Mingjun Lu, Jingwei Guo, Jinghong Wu, Chenyang Wang, Xiaoyue Zhu, Teng Ma

**Affiliations:** Cancer Research Center, Beijing Chest Hospital, Capital Medical University, Beijing Tuberculosis and Thoracic Tumor Research Institute, Beijing 101149, China

**Keywords:** DNA-PKcs, non-homologous end joining (NHEJ), ubiquitin-like modifications, PROTACs, cGAS-STING, radioresistance

## Abstract

Cells rely heavily on DNA repair networks to survive genomic damage. For repairing double-strand breaks, Non-Homologous End Joining (NHEJ) remains the primary pathway, which is largely controlled by the DNA-dependent protein kinase catalytic subunit (DNA-PKcs). Researchers have long studied how phosphorylation drives this kinase. However, recent data point to an important additional layer of control. Drawing on evidence accumulated over the past two decades, we propose a “Spatiotemporal Logic Circuit” model for DNA-PKcs regulation. In this model, SUMO-associated interactions may help stabilize synaptic assembly, HUWE1-mediated neddylation may facilitate kinase activation at Lys4007, and K48-linked ubiquitination—potentially involving RNF144A—may contribute to the turnover of persistent repair complexes. Importantly, we frame these UBL-mediated events within the broader autophosphorylation-driven conformational cycle of DNA-PKcs, which remains central to NHEJ progression. Additionally, we highlight the structural interface where activation and degradation signals may converge and the extraction barrier posed by the massive DNA-PKcs scaffold. From a translational perspective, we argue that the exceptional size of DNA-PKcs (~470 kDa) and its topological entrapment on DNA render it an unusually challenging PROTAC target—one that may require p97/VCP-assisted extraction before proteolysis can proceed. We also highlight the underappreciated risk that E3 ligase loss-of-function, already documented in BET-PROTAC resistance, may similarly undermine DNA-PKcs degrader strategies.

## 1. Introduction

### 1.1. The DNA-PK Holoenzyme and Its Dynamic Regulation by Autophosphorylation

Among the multiple pathways available to repair DNA double-strand breaks (DSBs), NHEJ is paradoxically both the fastest and the most error-prone—a trade-off that becomes clinically significant in rapidly dividing tumor cells under genotoxic stress [1,2]. The pathway hinges on the stepwise assembly of the DNA-PK holoenzyme: the ring-shaped Ku70/Ku80 heterodimer threads onto broken DNA ends within seconds of damage [3], after which the ~470 kDa catalytic subunit DNA-PKcs is recruited to form an initial long-range synaptic complex that physically tethers the two break ends [4].

What distinguishes DNA-PKcs from a passive scaffold is its capacity for self-regulation. Following stable assembly at the DSB, DNA-PKcs undergoes extensive autophosphorylation—most critically at the ABCDE and PQR clusters—a process characterized by Jette & Lees-Miller as essential for repair progression rather than merely for kinase activation per se [5,6,7]. The mechanical consequences of this event were later visualized using cryo-EM: both Ku and DNA-PKcs rotate outward by ~30°, exposing the DNA break and enabling downstream end-processing [8].

**Figure 1:**
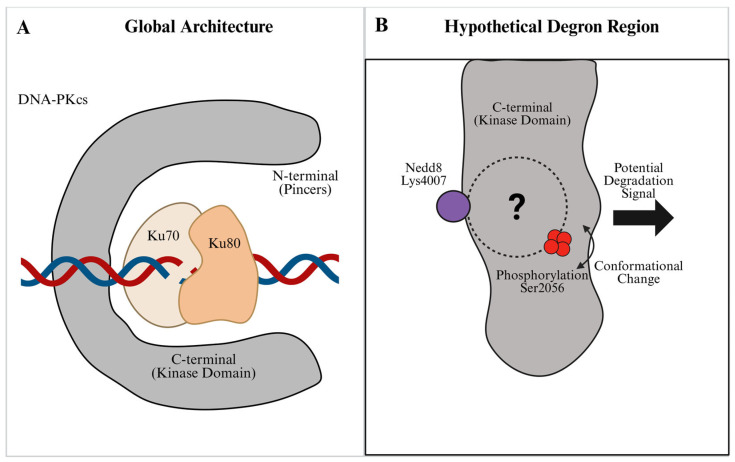
The structural landscape of UBL modifications on the DNA-PKcs scaffold. (**A**) Global Architecture: Schematic representation of the DNA-PK holoenzyme at a DNA double-strand break (DSB). The C-shaped DNA-PKcs catalytic subunit acts as a scaffold encompassing the Ku70/80 heterodimer bound to the broken DNA ends. (**B**) Hypothetical Degron Region: A localized view of the kinase domain featuring critical regulatory sites. Lys4007 (purple sphere) represents the neddylation site, while Ser2056 (red spheres) indicates the autophosphorylation cluster. Notably, autophosphorylation drives an outward conformational change (curved arrow) to remodel the complex. The dashed circle with a question mark represents the “Interface of Uncertainty,” a putative region where activation and potential degradation signals converge before triggering proteasomal turnover.

Therefore, rather than acting strictly as a static steric barrier on chromatin, the DNA-PKcs scaffold is actively regulated by its kinase activity [9]. It is within this highly dynamic environment that Ubiquitin-like (UBL) modifications—such as ubiquitination, neddylation, and SUMOylation—take effect. Rather than acting alone, these UBLs appear to form a higher-order regulatory network. They work alongside autophosphorylation to finely regulate the stability, activation, and final removal of the kinase, ensuring the repair process finishes correctly [10] (Figure 1A).

### 1.2. Ubiquitin-like Proteins: The Master Regulators

While phosphorylation has long been recognized as a key regulator of DNA-PKcs activity, in the past decade, accumulating evidence from over 20 studies has highlighted the pivotal role of covalent modification by ubiquitin-like proteins (UBLs). These modifications—including ubiquitination, neddylation, and SUMOylation—constitute a sophisticated code that governs the stability, localization, and interactome of DNA damage response (DDR) factors [10,11,12].

**Figure 2:**
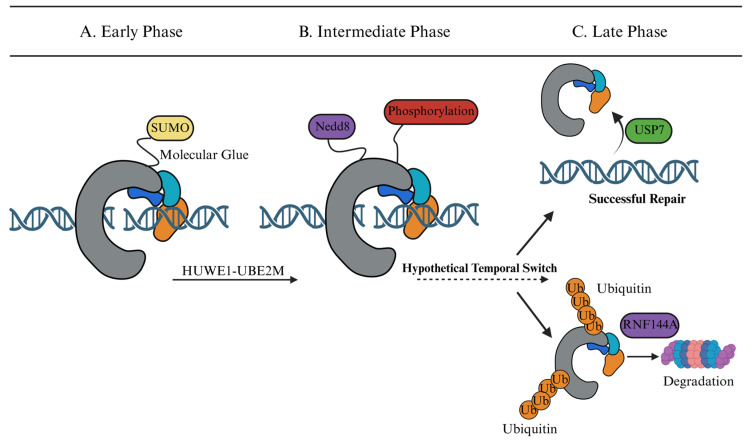
The Spatiotemporal “Logic Circuit” of DNA-PK regulation. The schematic summarizes a conceptual three-stage model of UBL-associated regulation of DNA-PKcs. (**A**) Early Phase: Upon DSB induction, SUMOylation acts as a molecular glue to stabilize the synaptic assembly. (**B**) Intermediate Phase: The HUWE1-UBE2M axis catalyzes neddylation, triggering kinase activation and autophosphorylation. A “Hypothetical Temporal Switch” (dashed arrow) marks the transition from the activation to the resolution phase, an area requiring further experimental validation. (**C**) Late Phase: A bifurcation point determines the fate of the complex. Successful repair yields a re-ligated, continuous DNA duplex and deubiquitinase-associated recycling/remodeling of repair factors. Conversely, stalled complexes undergo K48-linked polyubiquitination (e.g., via RNF144A), leading to 26S proteasomal degradation.

In particular, we examine how Guo et al.’s identification of HUWE1-mediated neddylation at Lys4007 reframed our understanding of how DNA-PKcs is activated, and how this insight connects to both ubiquitin-driven turnover and SUMO-mediated assembly, thereby providing a comprehensive overview of the “ubiquitin code” of DNA-PKcs [13]. Based on these findings, we propose a “Spatiotemporal Logic Circuit” model. In this framework, UBLs may act as more than simple chemical tags and can be conceptually viewed as contributing to the assembly, activation, and termination of the repair cycle (Figure 2) [14].

In the following sections, we first summarize how ubiquitination regulates DNA-PKcs turnover, then examine the activation role of neddylation, and finally discuss how SUMOylation organizes the assembly and disassembly of the repair complex.

## 2. Dynamic Turnover of DNA-PKcs via the Ubiquitin–Proteasome System

### 2.1. Ubiquitination as a Negative Regulator of DNA-PKcs

The ubiquitin–proteasome system (UPS) acts as a critical negative regulator of DNA-PKcs. While phosphorylation activates the kinase, ubiquitination acts as an “off-switch” to prevent the toxic accumulation of DNA-PKcs on chromatin, which could otherwise block DNA ends and impede alternative repair pathways [15,16]. Timely removal is therefore essential, which requires specific E3 ligases to coordinate degradation.

### 2.2. RNF144A as a Key Effector of DNA-PKcs Turnover

The E3 ligase RNF144A was identified as a key E3 ligase involved in damage-induced DNA-PKcs turnover. Ho et al. (2014) [15] demonstrated that upon severe DNA damage, p53 upregulates RNF144A expression. This ligase specifically targets DNA-PKcs for K48-linked polyubiquitination and subsequent proteasomal degradation. This mechanism effectively removes the pro-survival signals mediated by DNA-PKcs, thereby tipping the cellular fate toward apoptosis. Consistent with this, RNF144A depletion was shown to significantly reduce Caspase-3/7 activation and enhance clonogenic survival following doxorubicin treatment, confirming its pivotal role in the p53-dependent apoptotic axis [15].

### 2.3. Expanding the E3 Landscape: A Multilayered Regulatory Network

While RNF144A appears to function as an important effector of DNA-PKcs turnover, the regulation of the NHEJ machinery likely involves a broader network of E3 ligases. For example, RFWD3 has been identified as a regulator of homologous recombination (HR), where it ubiquitylates RPA and RAD51 to promote their timely removal from DNA damage sites and facilitate late HR progression [17]. Although this function is not NHEJ-specific, it illustrates how ubiquitin-dependent remodeling helps coordinate pathway-specific repair factor dynamics. Similarly, the HECT-domain E3 ligase TRIP12 plays a critical role in restraining excessive RNF168-dependent signaling, thereby fine-tuning the balance between ubiquitin-mediated recruitment and proteasomal degradation [18]. Although their direct action on DNA-PKcs remains to be fully mapped, these ligases collectively ensure that the repair complex is dynamically remodeled—specifically by orchestrating a balance between ubiquitin-mediated chromatin eviction, signaling attenuation, and subsequent proteasomal turnover—to prevent toxic persistence.

### 2.4. Clinical Implications: Drug-Induced Degradation for Radiosensitization

Recent studies have translated this mechanism into a therapeutic strategy for radiosensitization. Tsai et al. (2023) [19] revealed that treating cancer cells with Topoisomerase I inhibitors (e.g., Lipotecan) significantly upregulates RNF144A expression. The drug-induced RNF144A promotes the excessive ubiquitination and degradation of DNA-PKcs. This depletion of the core repair factor renders the tumor cells hypersensitive to radiotherapy. This study provides a therapeutic precedent suggesting that targeting the E3 ligase-DNA-PKcs axis may help overcome radiation resistance in solid tumors [19].

### 2.5. Indirect Stabilization: The USP7-HUWE1 Axis

While direct deubiquitination of DNA-PKcs is an area of active investigation (e.g., by BAP1), a well-established mechanism involves the stabilization of its upstream regulators. Khoronenkova & Dianov (2013) demonstrated that USP7 (HAUSP) functions as a specific deubiquitinase for Mule (HUWE1) [20]. USP7 removes poly-ubiquitin chains from HUWE1, preventing its proteasomal degradation and maintaining a sufficient pool of this E3 ligase in the nucleus [20]. Since HUWE1 is the key E3 ligase responsible for DNA-PKcs neddylation and activation (as discussed in Section 3), USP7 indirectly preserves the HUWE1-DNA-PKcs regulatory axis and may thereby support efficient NHEJ. Loss of USP7 destabilizes HUWE1 [20] and would therefore be expected to attenuate HUWE1-dependent activation of DNA-PKcs [13].

## 3. Dual Regulation of DNA-PKcs by Neddylation: Activation and Turnover

Protein neddylation, catalyzed by the sequential action of E1 (NAE), E2 (UBE2M/UBE2F), and E3 ligases, has emerged as a critical regulatory mechanism in the DNA damage response. Recent reviews highlight that UBE2M-mediated neddylation is particularly crucial for orchestrating the NHEJ pathway by modulating key factors like DNA-PKcs [21].

### 3.1. Direct Regulation: HUWE1-Mediated Neddylation Activates DNA-PKcs

Unlike the well-studied ubiquitination, the role of neddylation in regulating DNA-PKcs has only recently been elucidated. Guo et al. (2020) identified that DNA-PKcs is a direct target of NEDD8 modification [13]. Specifically, the E3 ligase HUWE1 was found to interact with and neddylate DNA-PKcs at its kinase domain, with Lys4007 being the primary conjugation site [13].

Mechanistically, this modification is crucial for the activation of DNA-PKcs. The study demonstrated that HUWE1-mediated neddylation promotes the autophosphorylation of DNA-PKcs at Ser2056, a key event required for NHEJ initiation. Consequently, inhibition of this pathway using the neddylation inhibitor MLN4924 or by depleting HUWE1 impairs Ser2056 phosphorylation and significantly reduces NHEJ efficiency, leading to the accumulation of DNA damage (indicated by sustained γH2AX foci). This finding establishes a direct link between the neddylation machinery and the core NHEJ complex [13].

### 3.2. Indirect Regulation: Neddylation-Dependent Control of Ku Heterodimer Removal

Beyond direct activation, neddylation exerts a profound indirect effect on NHEJ by controlling the dissociation of the Ku70/80 heterodimer from DNA ends. The ring-shaped structure of Ku poses a topological challenge: it must be actively removed to allow for downstream processing or to release the repaired DNA. Postow et al. (2008) demonstrated that the release of Ku80 from DNA is strictly dependent on its K48-linked polyubiquitination [22]. This ubiquitination is likely mediated by the SCF (Skp1-Cullin1-F-box) ubiquitin ligase complex, which requires neddylation of its Cullin-1 scaffold for enzymatic activity [23]. Therefore, inhibition of neddylation (e.g., by MLN4924) exerts a dual inhibitory effect on NHEJ: it prevents the activation of CRLs/SCF, causing Ku80 to fail to be ubiquitinated. This results in the retention (“trapping”) of Ku complexes on chromatin, physically blocking the access of downstream repair factors and preventing the resolution of DNA damage [22,24]. This dual role creates an apparent temporal duality: neddylation serves as both an “Ignition Key” for kinase activation (early phase) and a prerequisite for Ku80 removal (late phase). We propose that the transition between these opposing roles is likely governed by the accumulation of repair signals (e.g., phosphorylation thresholds) or the sequential recruitment of distinct E3 ligase complexes. However, it is important to acknowledge that a coordinated temporal switching mechanism between these pathways has not yet been experimentally demonstrated. Therefore, the proposed “temporal switch” should currently be considered a working hypothesis that warrants further investigation to clarify the precise kinetic handoffs between neddylation and ubiquitination in vivo.

## 4. SUMOylation: The “Molecular Glue” for Assembly and Nuclear Retention

In this review, we use the term “molecular glue” as a functional shorthand rather than as evidence for a fully defined scaffold mechanism. The current literature supports at least two SUMO-associated processes relevant to NHEJ: first, SUMO-linked stabilization of Ku70, which has been experimentally observed [25]; and second, poly-SUMO-dependent recruitment of SIM-containing factors, including RNF4, which likely contributes to the assembly of repair complexes. By contrast, direct SUMOylation sites on DNA-PKcs itself remain unresolved. Together, these observations provide a framework for understanding how SUMOylation may facilitate the rapid recruitment and nuclear retention of core NHEJ factors at DSBs [25,26,27]. This framework also aligns with emerging evidence that local biomolecular condensates formed through LLPS can help concentrate repair factors during the early DNA damage response [28,29].

### 4.1. Stabilization of the Ku Heterodimer via SUMO Shielding

The Ku70/80 heterodimer is the first responder to DNA damage, and its abundance in the nucleus determines the cell’s readiness for repair. Yurchenko et al. (2008) [25] showed that increased cellular SUMO is associated with marked stabilization of Ku70. However, the mechanistic basis of this effect remains incompletely resolved. One plausible explanation is that SUMOylation antagonizes ubiquitin-dependent turnover, either by competing for the same or adjacent lysine residues or by sterically limiting access of ubiquitin ligases. We therefore present SUMO–ubiquitin competition on Ku70 as a mechanistic model consistent with current observations, rather than as a directly demonstrated residue-level mechanism [25].

### 4.2. The SUMO-Ubiquitin Relay Mediated by STUbLs for Complex Disassembly

Repair complexes cannot stay on DNA forever; they must be removed to allow repair completion. This requires a “handover” from SUMO (assembly) to ubiquitin (removal). This process is mediated by RNF4, a STUbL. Galanty et al. (2012) [27] revealed that RNF4 contains SIMs that specifically recognize heavily SUMOylated proteins at DNA damage sites. RNF4 binds to these SUMO chains and then ubiquitylates the target proteins. This SUMO-ubiquitin crosstalk effectively translates a retention-favoring signal into a remodeling or removal signal, ensuring the timely turnover of repair factors and preventing their persistent occupation of repaired DNA. More broadly, such remodeling may also help reset end accessibility and thereby influence the transition between end protection and downstream processing [27].

## 5. The DNA-PK Logic Circuit: A Spatiotemporal Working Model

For conceptual clarity, we discuss these modifications according to their predominant functional associations; however, their biological effects are likely context-dependent and partially overlapping rather than strictly segregated. Although direct temporal sequencing has not yet been experimentally demonstrated, current evidence suggests that UBL modifications may occur in a coordinated order. More broadly, recent syntheses of the field have emphasized DNA-end synapsis as the organizing event that coordinates end bridging, processing, and ligation during NHEJ [30]. Three converging lines of evidence—the SUMOylation-dependent stabilization of Ku70 [25], HUWE1-mediated neddylation of DNA-PKcs at Lys4007 [13], and RNF144A-triggered K48-linked degradation [15]—suggest that UBL modifications do not act in isolation and may instead contribute to a partially ordered regulatory sequence. We propose a conceptual “Spatiotemporal Logic Circuit” as a working model in which distinct UBL modifications may help bias transitions between assembly, activation, turnover, and, potentially, repair-pathway-permissive states. At present, however, the precise temporal handoff between these pathways has not been directly demonstrated in vivo (Figure 2).

### 5.1. Phase 1: The Assembly Phase (Early)—SUMOylation as the Molecular Glue

The earliest molecular event at a DSB is not kinase activation but complex stabilization. Although the direct SUMOylation sites on DNA-PKcs itself remain to be mapped, modification of Ku70—and the consequent poly-SUMO-mediated recruitment of SIM-containing factors—may help stabilize the synaptic assembly before catalytic activity begins [25,26,27]. This ordering is consistent with single-molecule studies showing that NHEJ synapsis proceeds through at least two stages and that DNA-PK activity is required for transition to a short-range end synaptic complex [31]. This temporal priority makes biological sense: premature activation of DNA-PKcs before synapsis is complete would risk phosphorylating substrates out of context, potentially triggering erroneous end-processing.

### 5.2. Phase 2: The Activation Phase (Intermediate)

Once assembled, the circuit transitions to the firing phase. Current evidence supports a role for neddylation in promoting DNA-PKcs activation (Figure 2B). The recruitment of the HUWE1-UBE2M complex catalyzes the poly-neddylation of DNA-PKcs (specifically at Lys4007) [13]. This modification may function as an activation-promoting event, facilitating a conformational change that permits DNA-PKcs autophosphorylation at Ser2056 and activates its kinase activity. This temporal delay ensures that the kinase is only activated after proper synaptic assembly.

### 5.3. Phase 3: The Decision Phase (Late)—A Checkpoint for Repair Completion

How does a cell “know” when to stop assembling and start disassembling the repair complex? The answer may lie in a concentration-dependent threshold: as poly-SUMO chains accumulate on scaffold proteins beyond a critical density, they create a sufficiently high-affinity binding platform for the SIM domains of RNF4 [27]. Consistent with this view, Galanty et al. showed that RNF4 is recruited to SUMOylated substrates at DNA damage sites, supporting a SUMO-dependent recruitment model for RNF4 during DSB repair [27]. This recruitment event may represent an important transition point in the remodeling process, triggering RNF4 to ubiquitylate the complex and convert the signal from retention to extraction [27]. The final transition may function as a retention-dependent checkpoint during repair completion (Figure 2C) [32]. The system enters a bifurcation point determined by the duration of chromatin retention:

Two possible outcomes may follow at this decision node. First, if the repair is successfully completed, the repair machinery is recycled. Deubiquitinases may contribute to resetting the repair environment after damage, and USP7 is known to stabilize Mule/HUWE1, thereby indirectly influencing DNA damage signaling [20].

Alternatively, if the repair process is stalled or the complex persists on chromatin beyond a critical time threshold, a termination program is initiated. In this scenario, the E3 ligase RNF144A catalyzes K48-linked ubiquitination, which targets the stalled DNA-PKcs complex for proteasomal degradation [15].

### 5.4. A Hypothetical “Phospho-Degron” Mechanism: How RNF144A May Recognize Activated DNA-PKcs

While the neddylation site (Lys4007) and autophosphorylation clusters (ABCDE/PQR) have been structurally mapped [5,13], the spatial proximity of these regulatory regions also highlights a putative interface at which activation-associated and turnover-associated signals may converge (Figure 1B). A central unresolved question, therefore, remains: how does RNF144A discriminate between the activated form of DNA-PKcs and the quiescent enzyme?

We propose a “Phospho-Degron” model in which autophosphorylation at the ABCDE/PQR clusters induces the outward rotation of Ku and DNA-PKcs [8], thereby exposing cryptic surface regions that serve as recognition sites for RNF144A. This is conceptually analogous to phosphodegron-mediated substrate recognition in SCF ubiquitin ligase pathways, where phosphorylation of a defined motif directly recruits the F-box receptor [33]. Consistent with this model, Ho et al. established that RNF144A-mediated ubiquitination of DNA-PKcs is strictly dependent on upstream DNA damage signaling [15], suggesting that a damage-induced modification—most plausibly autophosphorylation—gates RNF144A recruitment.

However, two important caveats apply. First, a discrete phospho-degron on DNA-PKcs has not been structurally defined; cryo-EM capture of the RNF144A-DNA-PKcs complex is required to validate this model. Second, an indirect mechanism cannot be excluded: autophosphorylation-induced conformational changes may primarily destabilize the Ku70/80 interface [34], promoting DNA-PKcs dissociation from the break site [35] and thereby increasing its accessibility to E3 ligases, rather than generating a direct recruitment motif. Resolving which mechanism predominates—direct phospho-degron recognition versus dissociation-dependent ubiquitination—represents a tractable objective for future proximity-labeling or structural studies (see Section 6.4).

Thus, the UBL code may be viewed as a useful conceptual framework in which SUMOylation, neddylation, and ubiquitination preferentially contribute to assembly, activation, and turnover, respectively, while still allowing for context-dependent overlap.

### 5.5. Functional Implications for End Processing and Repair Pathway Choice

Beyond assembly, activation, and turnover, an important unresolved issue is how UBL-associated regulation of DNA-PKcs may influence end processing and thereby bias repair pathway utilization [4,36]. This question is biologically significant because DNA-PKcs does not act in isolation at DNA ends: its residence time, conformational state, and partner selection directly shape access of downstream processing factors, including Artemis, as well as factors that promote end resection and homologous recombination (HR) [4,36].

Artemis is particularly relevant in this context. As a nuclease activated in the DNA-PK complex, Artemis is essential for processing a subset of complex or blocked DNA ends and is also required for physiological end processing during V(D)J recombination [4,37]. In mammalian cells, Artemis-dependent trimming has further been linked to nucleosome-associated end processing during a subset of non-homologous end joining (NHEJ) events, especially when local chromatin organization imposes additional constraints on end accessibility [37]. These observations suggest that any PTM of DNA-PKcs that alters its persistence at DNA ends, kinase activation status, or interaction surface could secondarily modulate Artemis access or function, even if a direct UBL-Artemis relay has not yet been formally demonstrated [13,15,37].

The implications extend to repair pathway choice. Rather than a simple binary competition model, recent work supports a sequential framework in which DNA end protection, limited processing, and commitment to resection occur in ordered steps [36]. Within such a framework, PTMs that stabilize DNA-PKcs-containing end synapsis would be expected to favor NHEJ by maintaining end protection and restricting premature resection, whereas PTMs or associated signaling events that weaken DNA-PKcs retention or its interaction with end-associated factors may facilitate transition toward HR-permissive states in S/G2 cells [30,36]. Consistent with this view, SUMOylation of TIP60 at K430 has been shown to attenuate TIP60 interaction with DNA-PKcs in S-phase, thereby promoting HR and suppressing NHEJ-biased end protection [38]. Although this finding concerns a DNA-PKcs-interacting regulator rather than a UBL mark directly mapped on DNA-PKcs itself, it provides an important precedent that UBL-dependent remodeling of the DNA-PKcs interaction network can influence pathway choice [36,38].

Taken together, current evidence supports a model in which UBL-associated regulation of DNA-PKcs helps set the kinetic window for end protection, end processing, and complex clearance [13,15,36]. In turn, this window is likely to influence whether a broken chromosome end remains committed to NHEJ, progresses through Artemis-dependent processing, or becomes permissive for resection and HR [36,37]. Defining this transition more precisely will be important not only for understanding genome maintenance, but also for interpreting immunodeficiency, treatment-induced apoptosis, and therapeutic radiosensitization [4,15].

## 6. Conclusions and Perspectives

Although we discuss SUMOylation-, neddylation-, and ubiquitination-associated regulatory processes in a staged manner for conceptual clarity, available evidence does not support a rigid one-modification/one-function model. Taken together, current evidence suggests that SUMOylation, neddylation, and ubiquitination make distinct but interconnected contributions to the regulation of DNA-PKcs-containing repair complexes. Rather than representing a fully validated strict sequence, we present the “Spatiotemporal Logic Circuit” as a conceptual framework to organize current observations, guide future mechanistic studies, and help identify therapeutically targetable vulnerabilities in the UBL machinery.

### 6.1. Cancer Therapy and Metabolic Vulnerabilities

In cancer therapy, exploiting this UBL code provides new strategies for targeted degradation and radiosensitization. By targeting the upstream PTM machinery, researchers can impair DNA-PKcs function by blocking its assembly or activation. For instance, TAK-981 (subasumstat), a SUMO-activating enzyme inhibitor, has shown anti-tumor activity and may interfere with SUMO-dependent repair-associated processes [39]. Similarly, the neddylation inhibitor MLN4924 effectively impairs neddylation-dependent activation of DNA-PKcs [13,21].

**Figure 3:**
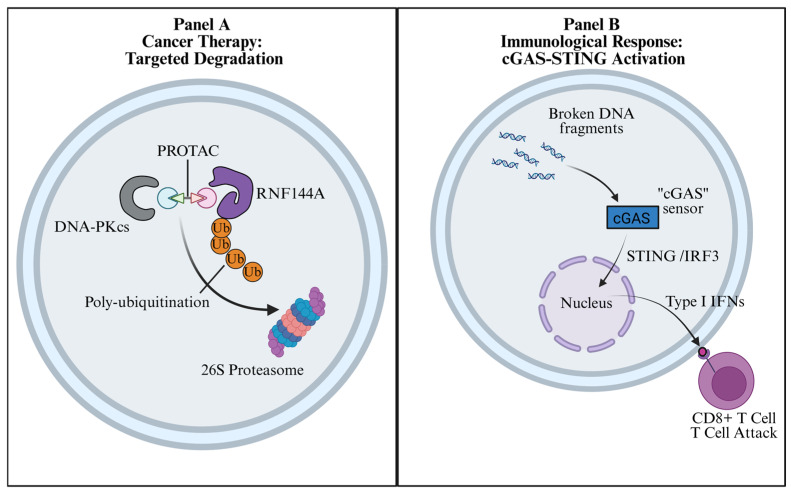
Therapeutic strategies targeting the Ubiquitin-like regulation of DNA-PKcs. The schematic illustrates two distinct clinical modalities exploiting the UBL code. (**A**) Cancer Therapy: Targeted Degradation. A PROTAC molecule bridges DNA-PKcs to an E3 ligase, enforcing poly-ubiquitination and subsequent destruction by the 26S proteasome to physically eliminate the repair machinery. (**B**) Immunological Response: cGAS-STING Activation. Pharmacological inhibition of DNA-PKcs prevents efficient repair, resulting in the leakage of broken DNA fragments into the cytosol. These fragments activate the cGAS-STING-IRF3 axis, driving the secretion of type I interferons (IFNs) and the subsequent recruitment of cytotoxic CD8+ T cells to the tumor microenvironment.

Beyond inhibition, targeted degradation aims to physically eliminate the DNA-PKcs scaffold (Figure 3A). In preclinical models, Topoisomerase I inhibitors (e.g., Lipotecan) have been shown to upregulate RNF144A expression, thereby promoting excessive ubiquitination and degradation of DNA-PKcs [19]. Furthermore, the physiological relevance of ubiquitin-mediated turnover is underscored by findings that the CRL4^DTL E3 ligase complex actively degrades DNA-PKcs; dysregulation of this pathway induces genomic instability and drives malignant transformation [40]. Targeting the RNF144A-associated degradation axis and the CRL4^DTL-dependent turnover pathway provides an opportunity to explore the emerging intersection between DNA repair signaling and cancer metabolism, as ubiquitination networks are critical nodes linking metabolic stress to DNA damage responses [41].

Developing Proteolysis Targeting Chimeras (PROTACs) for DNA-PKcs, inspired by successes with ATM degraders [42], remains a high-priority challenge. To contextualize this structural “Extraction Barrier,” Table 1 provides a comparative analysis of DNA-PKcs against other DDR kinases. Moreover, translating these mechanisms requires robust patient stratification. As summarized in Table 2, profiling the expression of specific E3 ligases and deubiquitinases—such as HUWE1 [13], USP7 [20], and RNF144A [15]—could provide a candidate biomarker framework for predicting radioresistance.

### 6.2. Immunological Responses: From V(D)J Recombination to Immunotherapy

Beyond direct cytotoxicity, manipulating the UBL regulation of DNA-PKcs has profound immunological consequences (Figure 3B) [4,37,47]. Physiologically, stable and properly regulated DNA-PKcs is required not only for end synapsis, but also for Artemis-dependent end processing during V(D)J recombination, underscoring how altered PTM control could affect lymphocyte development and responses to genotoxic therapy [4,19,37]. Pathologically, pharmacological inhibition of DNA-PKcs activity prevents efficient DSB resolution, leading to the accumulation of cytosolic DNA fragments. These fragments are subsequently sensed by the cGAS-STING pathway, triggering robust type I interferon signaling and recruiting cytotoxic CD8+ T cells [47]. Thus, targeting the UBL code provides a mechanistic rationale for synergistic combinations with immune checkpoint blockade (ICB) [47].

### 6.3. Neurological Diseases and Replication Stress

Potential implications of DNA-PKcs UBL regulation extend beyond cancer into neurobiology. Unlike proliferating cells, neurons are post-mitotic and rely predominantly on NHEJ to repair DSBs that accumulate over a lifetime of oxidative and metabolic stress. Efficient recycling of the DNA-PKcs complex is therefore likely to be important for maintaining neuronal genome integrity over time.

In Alzheimer’s disease, neurons exhibit chronically elevated γH2AX foci and impaired DNA-PKcs activity [48,49], findings that are broadly consistent with defective DSB repair capacity. However, a direct link between these observations and UBL-dependent termination or clearance of DNA-PKcs has not yet been established. In non-neuronal settings, DNA-PK inhibition has been linked to enhanced cGAS-STING activation [47]. Whether defective clearance of DNA-PKcs-containing repair complexes can promote a similar innate immune response in neuronal systems remains to be determined. Intriguingly, SUMOylation has been implicated in maintaining nuclear integrity and stress adaptation in neuronal or neuron-relevant contexts [50,51]. These observations raise the possibility that disturbed SUMO homeostasis could indirectly compromise genome maintenance in the nervous system, although its direct impact on DNA-PKcs-containing NHEJ assemblies remains unclear.

Furthermore, this discussion may also be relevant to replication-associated contexts in neural progenitor cells, where UbcH5c-dependent activation of DNA-PK has been reported to contribute to the resolution of replication-mediated DSBs [52].

### 6.4. Unresolved Questions and Emerging Technologies

Several critical questions remain to be resolved. First, in the “Lysine Code Question,” ubiquitin, NEDD8, and SUMO may compete for the same residues. A compelling precedent is observed in PML nuclear bodies, where poly-SUMO chains create a docking platform for the STUbL RNF4 to catalyze ubiquitination on distinct lysines [53]. Whether DNA-PKcs utilizes a similar “SUMO-primer, Ubiquitin-writer” cascade requires further investigation.

Second, overcoming PROTAC resistance represents a major hurdle. PROTAC resistance is frequently driven by the genomic loss of the recruited E3 ligase machinery [54,55]. Future degradation strategies targeting DNA-PKcs must recruit “essential” E3 ligases that cancer cells cannot afford to lose [56].

Finally, to bridge the gap between static structures and dynamic regulation, future studies should employ emerging technologies: Proximity Labeling (e.g., BioID/APEX2) to map transient E3-substrate interactions [57]; Single-Molecule Imaging for real-time tracking of PTM dynamics [58]; and in situ Cryo-ET to capture the native conformation of the DNA-PKcs extraction intermediates [59]. These approaches will be important for translating this molecular framework into clinical applications.

## Figures and Tables

**Table 1:** Comparative analysis of targeted degradation challenges among PIKK family kinases.

Feature	DNA-PKcs (Structural Scaffold and Kinase)	ATM	ATR
**Primary Role**	Structural Scaffold & Kinase: Forms a synaptic complex at DNA ends and helps bridge broken DNA termini [4].	Signal Transducer: Recruited to DSB ends (via MRN) primarily to initiate signaling; activation involves dimer dissociation [43].	Signal Transducer: Engages RPA-coated ssDNA and coordinates replication stress signaling.
**Protein Stability & Turnover**	Damage-Responsive Turnover: Persistent DNA-PKcs complexes may require active removal during late repair or damage-induced remodeling. Stability is dynamically influenced by the RNF144A axis [15].	Predominantly Activation-State Controlled: ATM regulation is driven mainly by activation-state changes rather than overt damage-induced bulk degradation; activation is conformational (dimer-to-monomer) rather than abundance-driven [44].	Relatively Stable Signaling Kinase: ATR abundance is not usually discussed as a rapidly turned-over parameter, although it can be experimentally reduced by degrader strategies [45].
**Degradation Challenge**	High (Topological/Steric Barrier): The topological architecture of DNA-PKcs suggests that active extraction mechanisms may be required prior to proteolysis; p97/VCP-mediated removal of sterically trapped Ku70/80 provides a relevant mechanistic precedent [46].	Moderate: ATM is dynamically associated with chromatin and is not thought to face the same topological extraction barrier as DNA-PKcs.	Moderate: ATR may be subject to ubiquitin-dependent regulation, but it generally lacks the topological entrapment characteristic of DNA-PKcs.
**Proteasomal Regulation**	Functionally Linked: Degradation may contribute to termination-associated clearance of stalled DNA-PKcs complexes, potentially involving RNF144A and extraction mechanisms inferred from p97/VCP-dependent precedents.	Fine-Tuning: Ubiquitin signaling appears to regulate ATM recruitment and signaling dynamics more prominently than bulk degradation.	Context-Dependent: ATR abundance and stability may be modulated under selected stress contexts, rather than through constitutive bulk turnover.
**Therapeutic Opportunity**	Immunogenic Potential: DNA-PK inhibition can enhance cGAS-STING activation in selected contexts, suggesting that disruption of DNA-PKcs-dependent repair may increase tumor immunogenicity [47].	Combination Potential: ATM loss or inhibition may create therapeutic opportunities in selected synthetic-lethal settings, including combinations with PARP- or ATR-directed strategies.	Replication-Stress Vulnerability: ATR-directed strategies may be particularly relevant in tumors characterized by high replication stress.

**Table 2:** Mechanistically Implicated UBL Enzymes Requiring Clinical Validation.

Biomarker	Function in DNA-PK Regulation	Context	Clinical Association	Evidence Level	Reference
UBE2M (High)	E2 for neddylation; implicated in activation-associated neddylation pathways	General/candidate biomarker	Associated with an adverse prognosis in selected cancers	Level 3	Zhou et al. [21]
HUWE1 (High)	E3 for DNA-PKcs neddylation; supports activation	General/mechanistic	Associated with reduced sensitivity to DNA damage-based therapy	Level 3	Guo et al. [13]
USP7 (High)	DUB; stabilizes HUWE1 and indirectly supports DNA-PKcs regulation	General	Potentially associated with reduced sensitivity to DNA damage-based therapy	Level 3	Khoronenkova et al. [20]
RNF144A (Baseline Low)	E3 for DNA-PKcs degradation	Baseline low	Low baseline expression may be associated with impaired apoptotic response and unfavorable outcome	Level 3	Ho et al. [15]
RNF144A (Drug-Induced High)	E3 for DNA-PKcs degradation	Drug-induced high	Associated with radiosensitization in drug-induced settings	Level 3	Tsai et al. [19]

Note: Level 3: Level 3 indicates support from strong mechanistic evidence (e.g., genetic knockdown/overexpression studies) and limited clinical correlation. Further independent validation is required.

## Data Availability

Not applicable.

## Abbreviations

DDR	DNA Damage Response
DSB	DNA Double-Strand Break
NHEJ	Non-Homologous End Joining
HR	Homologous Recombination
DNA-PKcs	DNA-dependent Protein Kinase catalytic subunit
UBL	Ubiquitin-like protein/modification
UPS	Ubiquitin–Proteasome System
SUMO	Small Ubiquitin-like Modifier
SIM	SUMO-Interacting Motif
STUbL	SUMO-Targeted Ubiquitin Ligase
DUB	Deubiquitinase
CRL	Cullin-RING Ligase
PROTAC	Proteolysis Targeting Chimera
LLPS	Liquid–Liquid Phase Separation
ICB	Immune Checkpoint Blockade
V(D)J	Variable–Diversity–Joining (recombination)
